# Income Differences in the Association Between Medical-Preventive Integration KAP and Depressive Symptoms: A Cross-Sectional Study Among Chinese Healthcare Workers

**DOI:** 10.3390/healthcare14131832

**Published:** 2026-06-24

**Authors:** Ting Wang, Qiaosheng Li, Xiaoyu Fang, Yujie Shang, Weiyan Jian, Bing Li

**Affiliations:** 1 National Center for Infectious Diseases, Beijing Ditan Hospital, Capital Medical University, Beijing 100015, China; 2111110172@bjmu.edu.cn; 2 Department of Health Policy and Management, School of Public Health, Peking University, Beijing 100191, China; qshli@bjmu.edu.cn; 3 Department of Social Medicine and Health Management, School of Public Health, Cheeloo College of Medicine, Shandong University, Jinan 250012, China; sddxgwfxy@163.com; 4 School of Artificial Intelligence, Beijing University of Posts and Telecommunications, Beijing 100876, China; 2516621848@bupt.edu.cn

**Keywords:** Knowledge-Attitude-Practice (KAP), medical-preventive integration, depressive symptoms, work stress, income, healthcare workers

## Abstract

**Highlights:**

**What are the main findings?**

- Medical-preventive integration KAP was significantly associated with depressive symptoms among healthcare workers.
- Knowledge and attitude were protective factors, whereas practice was associated with higher depressive symptoms.

**What are the implications of the main findings?**

- Work stress and socioeconomic differences should be considered in designing interventions for depressive symptoms among healthcare workers.
- Strengthening workload allocation and institutional support may help improve occupational mental health in healthcare reform contexts.

**Abstract:**

**Objective:** To examine the relationship between Knowledge, Attitude, and Practice (KAP) regarding medical-preventive integration and depressive symptoms among Chinese healthcare workers, placing special emphasis on the moderating association of monthly income, while adjusting for work stress as an important covariate. **Methods:** This study analyzed data from a 2024 cross-sectional survey conducted in two provinces of China, encompassing a total of 5908 healthcare workers. Descriptive statistics were employed to examine the demographic and professional characteristics of the participants. Multiple linear regression was used to investigate the direct associations between medical-preventive integration knowledge, attitude, practice, and depressive symptoms. Furthermore, while moderation analysis was used to assess the interactive association of monthly income in this relationship. **Results:** The findings demonstrate a significant link between medical-preventive integration knowledge, attitude, and practice (KAP) and depressive symptoms, with knowledge (B = −0.074, 95% CI = −0.134, −0.015) and attitude (B = −0.467, 95% CI = −0.531, −0.403) showing a negative association with depressive symptoms, while practice (B = 0.648, 95% CI = 0.527, 0.770) was positively associated with depressive symptoms. Work stress was positively associated with both practice engagement and depressive symptoms. Additionally, higher income amplified the inverse associations of knowledge (B = −0.040, 95% CI = −0.061, −0.019) and attitude (B = −0.028, 95% CI = −0.046, −0.012) with depressive symptoms and strengthened the positive association between practice (B = 0.066, 95% CI = 0.022, 0.109) and depressive symptoms, as indicated by significant interaction association. **Conclusions:** Our findings highlight a complex link between KAP patterns and depressive symptoms, with work stress as a significant correlate and income as a moderator. Healthcare workers with higher income and those with supportive knowledge and attitude show a lower probability of experiencing depressive symptoms, whereas greater behavioral engagement is associated with increased depressive symptoms, particularly among those with higher income. There is an urgent requirement to establish targeted interventions, optimizing support and resources, to decrease susceptibility to depressive symptoms among healthcare workers, particularly those with higher income and higher levels of behavioral engagement.

## 1. Introduction

In recent years, China has implemented a series of reforms aimed at integrating its historically fragmented healthcare delivery system to improve overall service efficiency [1]. Guided by the Healthy China 2030 strategy, the national healthcare system is undergoing a profound transformation, transitioning from a treatment-centered to an integrated, prevention-oriented model [2,3]. To better address the population’s growing demand for high-quality health services, China has initiated medical-preventive integration reforms aimed at enhancing the efficiency of healthcare delivery while providing comprehensive, life-course health services for the population [4]. Simultaneously, it has substantially reshaped the roles and responsibilities of healthcare professionals. In addition to traditional clinical duties, medical personnel are now increasingly tasked with preventive and public health activities, resulting in higher role complexity and increased workload [5]. Against this backdrop, the mental health of healthcare providers has emerged as a critical concern, as the evolving demands of integrated care may pose novel psychological challenges.

Accompanying the growing recognition of occupational health as a pivotal component of public health is increased attention to the mental well-being of healthcare personnel. A substantial body of research indicates that the prevalence of depressive symptoms among medical staff is markedly higher than that observed in the general population [6,7,8]. Beyond impairing individual occupational functioning and performance, depressive symptoms among healthcare providers have been linked to an elevated probability of medical errors, reduced job satisfaction, and increased turnover intentions, thereby posing a potential threat to the stability and quality of healthcare service delivery [9,10]. Amid the ongoing implementation of medical-preventive integration, healthcare personnel are confronted with emerging work-related stressors. While standard sociodemographic attributes (e.g., gender, age, and professional title) function as important static covariates linked to depressive symptoms [11,12], monthly income warrants distinct theoretical attention as a dynamic moderator. Grounded in the Effort-Reward Imbalance (ERI) framework, income represents the primary tangible ‘reward’ that directly interacts with reform-driven workloads. Insufficient understanding of, or poor adaptation to, the reform’s policies may exacerbate occupational pressure and increase the probability of psychological distress. Identifying modifiable factors associated with depressive symptoms in healthcare providers is therefore crucial for ensuring the sustainable functioning of healthcare systems and safeguarding service quality.

Knowledge, attitude, and practice (KAP) regarding medical-preventive integration may exert a significant influence on the psychological health of healthcare personnel. According to the KAP framework, adequate policy knowledge and positive attitude can enhance role clarity and professional identity, which may mitigate the risk of psychological distress [13,14]. Conversely, insufficient understanding or lower levels of attitudinal alignment may increase adaptation pressure and emotional burden. Viewed through the lens of the Job Demands-Resources (JD-R) model, lower KAP levels may constitute a deficit in psychological resources, heightening the perception of job demands as stressful [15]. As sustained work stress is a well-established risk factor for depression, it may be closely intertwined with KAP and depressive symptoms [16]. Furthermore, income, as a key indicator of socioeconomic status, shapes individual resource reserves and coping capacity [17]. Under comparable levels of occupational stress, individuals with higher income may possess greater buffering capacity, whereas those with lower income may be more susceptible to psychological problems, indicating a potential moderating role of income in the associations among KAP, work stress, and depressive symptoms [18]. Despite these theoretical rationales, empirical studies systematically examining the interplay of KAP, work stress, and income in relation to depressive symptoms within the context of medical-preventive integration remain scarce. Drawing on the JD-R model, we conceptualize knowledge and attitude as psychological resources that may buffer depressive symptoms, while practice engagement represents an additional job demand that may increase psychological strain. This resource-demand distinction guides our hypothesis development and model interpretation.

Therefore, this study aimed to examine the association between KAP regarding medical-preventive integration and depressive symptoms among Chinese healthcare workers. Using data from a large cross-sectional survey conducted in two provinces of eastern and western China in 2024, we specifically assessed the moderating association of income, while including work stress as an important covariate.

## 2. Methods

### 2.1. Data Sources and Sample Composition

This study was conducted in two provinces located in eastern and western China, where pilot programs on medical-preventive integration were being implemented. Data were collected in December 2024.

The sample size was calculated prior to data collection based on the total number of healthcare professionals employed in pilot medical institutions across the two provinces. An a priori power analysis was conducted using G*Power (Version 3.1) to determine the required sample size for multiple linear regression. Assuming a small effect size (ƒ^2^ = 0.02), a significance level (alpha) of 0.05, and up to 25 predictors, a minimum of 719 participants was required to achieve a statistical power of 80%. Our final analytical sample of 5908 participants provides an exceptional statistical power (1 − *β* > 0.999) to detect even a highly conservative effect size, effectively eliminating the risk of Type II errors. A multistage stratified random sampling method was employed to enhance representativeness and minimize selection bias. First, medical institutions participating in the pilot program were stratified according to hospital level (primary, secondary, and tertiary). Within each stratum, institutions were selected using a computer-generated random number sequence. Subsequently, eligible healthcare professionals were randomly sampled from staff rosters provided by the human resources departments of the selected institutions. Proportional allocation was applied to ensure appropriate representation across hospital levels.

Eligible participants were healthcare professionals engaged in medical–preventive integration-related work. The inclusion criteria were as follows: (1) having at least one year of work experience; (2) being a formally employed staff member of the institution; (3) providing voluntary informed consent to participate in the study. The exclusion criteria were: (1) not being formally employed by the institution (e.g., visiting staff, interns, postgraduate students, or residents in standardized training programs); (2) refusal to participate; (3) the variables of interest in this study were missing. After meticulous screening, a total of 5908 participants were ultimately included in this study. Informed broad consent was obtained from all participants prior to data collection, and all data were anonymized to ensure confidentiality. The questionnaire was administered through an online survey platform.

Data were collected using a structured, self-administered questionnaire distributed via a secure online survey platform. To ensure data quality, several measures were implemented: (1) each device/IP address was permitted to submit the questionnaire only once; (2) mandatory response fields were set for key variables; (3) logical consistency checks were embedded within the questionnaire; and (4) incomplete or inconsistent responses were excluded during data cleaning. All responses were anonymized prior to analysis to ensure confidentiality. The final analytic sample of 5908 participants exceeded the calculated minimum requirement, ensuring adequate statistical power for the planned analyses.

### 2.2. Main Outcome Measures: Depressive Symptoms

The CES-D-10 questionnaire, developed by Radloff, stands as one of the predominant self-assessment tools for depressive symptoms. Scale Revised (CES-D-10), a self-report measure composed of 10 items assessing symptoms of depression in the last week, was derived from the original 20-item CES-D [19]. Specifics of the 10-item CES-D scale can be found in Table S1.

The CES-D-10 comprises three items assessing depressive affect (feeling depressed, fearful, and lonely), five items reflecting somatic symptoms (being bothered by minor matters, difficulty concentrating, reduced activity, restless sleep, and low energy), and two items measuring positive affect (hopefulness about the future and happiness). Each item is rated on a four-point Likert scale ranging from “rarely or none of the time” (score of 0) to “all of the time” (score of 3). Prior to calculating the continuous total score, the two positive affect items were reverse-coded (0 = 3, 1 = 2, 2 = 1, 3 = 0). Within the CES-D-10 framework, depressive symptoms were gauged on a scale from 0 to 30, with elevated scores indicating more severe symptoms [20]. In the primary statistical analyses of this study, depressive symptoms were treated strictly as a continuous outcome variable based on this total score. In the present study, the scale demonstrated good internal consistency, with a Cronbach’s alpha of 0.831.

### 2.3. Main Measures of Independent Variables

#### 2.3.1. Medical-Preventive Integration Knowledge, Attitude, Practice (KAP)

The Medical-Preventive Integration Knowledge-Attitude-Practice (KAP) Questionnaire was developed by the research team based on the Knowledge-Attitude-Practice theoretical framework and relevant policy documents on medical-preventive integration in China, with initial items generated through a comprehensive literature review and expert consultation, followed by a pilot survey to refine the wording and structural validity of the instrument.

The Medical-Preventive Integration KAP scale comprises three subscales: knowledge, attitude, and practice. The knowledge dimension (4 items) covers concepts, functions, implementation measures, and system construction of medical-preventive integration. The attitude dimension (4 items) includes perceptions toward reform, training, information system development, and mutual recognition of results. The practice dimension (2 items) assesses the frequency of work-related practice and training participation. The full text and specific details of the questionnaire items are provided in Table S2. Each item is rated on a five-point Likert scale ranging from “strongly disagree” (score of 1) to “strongly agree” (score of 5). Higher scores indicate greater knowledge, more positive attitudes, and more active engagement in medical-preventive integration practice. In the present study, the scale demonstrated excellent internal consistency, with Cronbach’s alpha values of 0.97 for the knowledge subscale, 0.96 for the attitude subscale, and 0.90 for the practice subscale (overall scale α = 0.93). To evaluate its structural validity, Confirmatory Factor Analysis (CFA) was conducted on the total sample (N = 5908). The 3-factor model exhibited an excellent fit to the empirical data: χ^2^/dƒ = 0.674, CFI = 0.970, TLI = 0.942, and RMSEA = 0.010. Furthermore, all standardized factor loadings significantly exceeded the 0.40 threshold, ranging from 0.428 to 0.749 (*p* < 0.001), confirming robust structural validity for the scale.

#### 2.3.2. Work Stress

The Work Stress Scale, developed by House and Rizzo in 1972, was used to assess the psychological tension and stress perceived by medical staff in the context of medical-preventive integration [21]. The scale consists of seven items (e.g., “I work under a great deal of tension”) and is rated using a five-point Likert scale ranging from “strongly disagree” (score of 1) to “strongly agree” (score of 5). Higher total scores indicate greater perceived work stress. This scale has been widely applied and adapted by Chinese scholars, demonstrating good reliability and general applicability across settings [20]. In the present study, Cronbach’s alpha of this scale was 0.930. The scale items are presented in Table S3.

#### 2.3.3. Income

Monthly income of medical staff was treated as a moderating variable in this study. In the questionnaire design, monthly income was categorized into approximately ten groups at intervals of 2000 RMB, ranging from “below 4000 RMB” to “20,000 RMB and above.” Higher categories represent higher levels of monthly income. For statistical analysis, the midpoint of each income category was assigned and treated as a continuous variable. For the open-ended highest income category (“20,000 RMB and above”), a value of 21,000 RMB was assigned based on the width of the adjacent income interval.

### 2.4. Potential Confounders

We examined the distribution of key demographic and professional characteristics within the study sample. These control variables included: gender (male, female); age group (18–29, 30–39, 40–49, 50–59, ≥60 years); ethnicity (Han Chinese, ethnic minorities); political status (Communist Party of China member, probationary CPC member, Communist Youth League member, member of a democratic party, non-partisan individual, general public); education level (secondary vocational school or below, college diploma, bachelor’s degree, master’s degree, doctoral degree); professional title (none, junior, intermediate, associate senior, senior); and hospital level (tertiary hospital, secondary hospital, primary/community hospital). Existing literature indicates a correlation between depressive symptoms and various demographic, socioeconomic, and health attributes [11,12].

### 2.5. Statistical Analysis

Depressive symptoms were the dependent variable, and medical-preventive integration knowledge, attitude, and practice were included as the independent variables. Descriptive statistics were first performed to summarize the distribution of demographic and professional characteristics of the study participants. Multiple linear regression analysis was conducted to examine the associations between medical-preventive integration knowledge, attitude, practice, and depressive symptoms. Work stress was included as a covariate in all regression models to adjust for its potential confounding association. In addition, monthly income was treated as a moderating variable in the regression models to assess potential moderation association. The association of confounding factors mentioned above on the outcome variables was controlled in the multiple linear regression model. Stata version 18.0 was used for data analysis. The statistical significance level was set at *p* < 0.05. Unstandardized regression coefficients (B) were reported for all hierarchical linear models.

## 3. Results

### 3.1. Descriptive Statistical Analysis

The descriptive analysis of the main variables in our study is presented in Table 1. The mean scores for knowledge, attitude, and practice regarding medical-preventive integration were 12.32 (SD = 3.60), 14.82 (SD = 3.03), and 5.93 (SD = 1.73), respectively. The respondents’ average scores for depressive symptoms were 9.36 (SD = 6.10). The mean work stress score was 23.46 (SD = 6.33), and the average monthly income was 10.33 thousand RMB (SD = 5.35). Among 5908 respondents, females (*n* = 3315, 56.11%), individuals aged 30–39 years (*n* = 2845, 48.16%), married individuals (*n* = 4797, 81.20%), Han Chinese (*n* = 5816, 98.44%), non-affiliated individuals (*n* = 2690, 45.53%), those with a bachelor’s degree (*n* = 3263, 55.23%), individuals with an intermediate professional title (*n* = 2100, 35.55%), and those working in tertiary hospitals (*n* = 3977, 67.32%) accounted for the majority.

### 3.2. Multiple Linear Regression Analysis

We first estimated a base model including only covariates (see Table S4). Table 2 presents the results of the multiple linear regression analysis assessing the factors associated with depressive symptoms. In Model 1, with confounding variables controlled, knowledge, attitude, and practice regarding medical-preventive integration were all significantly associated with depressive symptoms. Specifically, higher levels of knowledge (B = −0.074, 95% CI: −0.134, −0.015) and more positive attitudes (B = −0.467, 95% CI: −0.531, −0.403) were associated with fewer depressive symptoms. In contrast, greater behavioral involvement in medical-preventive integration was significantly associated with increased depressive symptoms (B = 0.648, 95% CI: 0.527, 0.770). In Model 2, work stress was positively associated with depressive symptoms (B = 0.206, 95% CI: 0.044, 0.368).

### 3.3. The Interactive Association of Income

**Figure 1:**
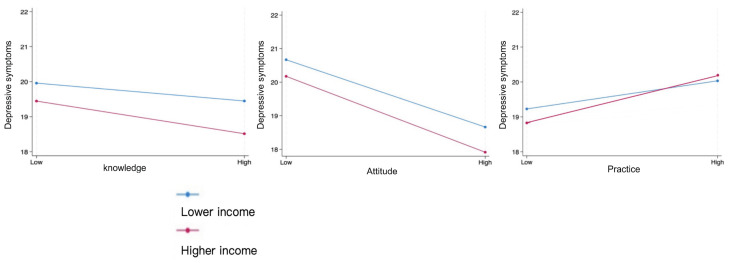
Moderating association of monthly income on the associations between knowledge, attitude, practice and depressive symptoms. Note. Lines indicate predicted depressive symptom scores at low and high levels of each KAP dimension for lower and higher income groups. Monthly income was entered as a continuous moderator in the regression model, and the plotted values represent lower and higher levels of income used for simple-slope visualization rather than actual monthly income values (Model 3).

Table 2 (Model 3) and Figure 1 present the regression results after introducing the interaction terms between monthly income and medical-prevention integration knowledge, attitude, and practice on depressive symptoms. Significant interaction associations were observed for knowledge*income (B = −0.040; 95% CI: −0.061, −0.019), attitude*income (B = −0.028; 95% CI: −0.046, −0.012), and practice*income (B = 0.066; 95% CI: 0.022, 0.109). Specifically, as income increases, higher levels of knowledge and more positive attitudes are associated with a lower probability of depressive symptoms, whereas the positive association between behavioral engagement in medical-preventive integration and depressive symptoms becomes stronger.

Compared with lower-income healthcare personnel, those with higher income exhibit a stronger inverse association between knowledge and attitude toward medical-preventive integration and depressive symptoms, as well as a stronger positive association between behavioral engagement and depressive symptoms.

## 4. Discussion

This study focused on depressive symptoms among medical staff and examined the relationship between KAP regarding medical-preventive integration and depressive symptoms, and further examined the moderating role of monthly income, with work stress included as an important covariate. In this study, we found that KAP of medical-preventive integration was significantly associated with depressive symptoms. In addition, the results of this study reveal that work stress was independently and robustly associated with elevated depressive symptoms, while monthly income may serve as a moderating factor in this relationship.

Our investigation reveals a complex and dimension-specific association between KAP concerning medical-preventive integration and depressive symptoms, highlighting the divergent psychological implications of cognitive-attitudinal alignment versus practical engagement. This aligns with the continuum of findings extant in prior research [22]. Specifically, higher levels of knowledge and more positive attitudes were inversely associated with depressive symptoms, whereas greater behavioral involvement was positively correlated with these outcomes. This divergence underscores the critical distinction between the psychological resources afforded by conceptual understanding and the heightened job demands entailed by practical implementation. From the perspective of KAP theory and cognitive appraisal frameworks, comprehensive knowledge regarding medical-preventive integration may serve to reduce uncertainty surrounding policy reforms and clarify role expectations, thereby enhancing psychological safety and resilience [23]. Concurrently, favorable attitudes toward reform initiatives may foster a sense of professional purpose, organizational identification, and alignment with institutional objectives, which operate as psychological resources that buffer against emotional distress [24]. Previous research has indicated that cognitive comprehension and positive professional perceptions constitute protective factors for mental health among healthcare personnel [25]. While knowledge and attitude function as psychological resources, active participation in medical-preventive integration amplifies job demands, encompassing expanded responsibilities, administrative obligations, and potential role conflicts. When these demands outstrip the available personal and organizational resources, the resulting psychological strain may increase vulnerability to depressive symptomatology [26]. This finding explains the paradox whereby practical engagement, despite being underpinned by supportive knowledge and attitude, is nonetheless positively associated with higher levels of mental health depressive symptoms. Collectively, these findings indicate that the mental health consequences of reform participation are multifactorial and context-dependent, contingent upon the balance between psychological resources and occupational demands. From a theoretical perspective, these findings extend the application of the KAP framework beyond behavioral outcomes by demonstrating its relevance to mental health in the context of healthcare reform. The results also provide empirical support for the Job Demands–Resources (JD-R) model, suggesting that knowledge and attitude may function as psychological resources, whereas intensive practice engagement may operate as a job demand with potential psychological costs. This highlights the importance of considering different KAP dimensions separately when examining occupational well-being. Interventions aimed at optimizing workload allocation, clarifying role expectations, and enhancing organizational support may be instrumental in mitigating the adverse psychological impact of practical reform engagement, while simultaneously preserving the protective benefits conferred by knowledge acquisition and attitudinal alignment.

More precisely, healthcare personnel exhibiting higher engagement in medical-preventive integration practice demonstrate an increased susceptibility to perceived work stress, which, in turn, contributes to elevated levels of depressive symptoms. This finding is largely consistent with the extant literature on occupational stress and mental health outcomes [27,28]. From the perspective of role overload and effort-reward imbalance theories, the accumulation of job demands-encompassing administrative responsibilities, health education, chronic disease management, information reporting, and cross-sector coordination-may substantially intensify perceived pressure, thereby heightening the likelihood of psychological strain [29]. Prior studies have consistently shown that excessive workload in the absence of adequate organizational and psychosocial support exacerbates occupational stress, which serves as a critical determinant of depressive symptomatology among healthcare personnel [30]. Furthermore, the relationship between behavioral engagement in medical-preventive integration and mental health outcomes appears to be intricately contingent upon the availability of psychological and organizational resources. Our results confirm that work stress is strongly correlated with both practice engagement and depressive symptoms, supporting the hypothesis that increased professional involvement—despite its alignment with organizational objectives—may inadvertently compromise mental well-being. Nevertheless, due to the cross-sectional design, causal interpretations should be made with caution, and the observed associations are correlational. The present findings suggest several directions for future research. Given that the current analysis is based on cross-sectional data, longitudinal studies are needed to clarify the temporal relationships among KAP, work stress, and depressive symptoms. Future research may also explore additional organizational and psychosocial factors, such as leadership support, workplace climate, and professional identity, that could influence these associations. Consequently, these findings underscore the importance of targeted interventions aimed at optimizing workload allocation, clarifying role expectations, and enhancing organizational support systems. Such strategies hold considerable promise for mitigating perceived work stress and, in turn, alleviating depressive symptomatology among healthcare personnel engaged in the multifaceted demands of medical-preventive integration.

An additional noteworthy finding from our investigation is the moderating role of monthly income in the association between KAP and depressive symptoms, suggesting that the psychological impact of medical-preventive integration is contingent upon socioeconomic resources. This observation diverges from studies that treat reform-related stressors as uniformly affecting healthcare personnel, instead highlighting the heterogeneity of mental health outcomes across different income strata. Specifically, individuals with higher income levels appeared to be better equipped to derive knowledge and positive attitudes toward reform into psychological benefits, likely due to greater access to coping resources, social support networks, and job autonomy, which may enhance psychological resources but do not fully offset the increased job demands associated with behavioral engagement [16]. This pattern can be interpreted through the lens of the Conservation of Resources (COR) theory, which posits that individuals with greater resource reserves are more stress-resilient and less susceptible to resource loss. Under relatively favorable economic conditions, engagement in reform initiatives-manifested through knowledge acquisition and attitudinal alignment-may enhance psychological well-being. However, our results indicate that higher income also strengthens the positive association between behavioral engagement and depressive symptoms. One possible explanation is that healthcare workers with higher income may be more likely to hold positions associated with greater responsibilities and performance expectations, thereby being exposed to elevated job demands when engaging in medical-preventive integration practices [31]. These findings underscore that the mental health consequences of reform participation are shaped by a complex interplay between socioeconomic resources and job demands, rather than a simple buffering mechanism. While higher income enhances the protective association of knowledge and attitude, it simultaneously amplifies the probability of psychological distress associated with intensive behavioral engagement. Hence, targeted psychological interventions and incentive policies that prioritize lower-income medical staff engaged in intensive reform-related tasks may hold considerable promise for mitigating depressive symptoms and fostering equitable occupational well-being. From a practical standpoint, these findings suggest that healthcare organizations should not only promote engagement in medical-preventive integration but also ensure that adequate organizational resources accompany implementation efforts. Strategies such as workload redistribution, role clarification, stress management programs, and supportive supervision may help reduce the unintended psychological burden associated with intensive reform participation.

### Limitations

There are several limitations of the current study that warrant discussion, and the results should be interpreted with some caution. Firstly, data on depressive symptoms and work stress were collected through self-reported measures, which may have introduced some degree of response bias. Secondly, our cross-sectional design limits causal inference regarding the associations among KAP, work stress, and depressive symptoms; reverse causality and unmeasured confounding (e.g., organizational climate factors or other psychological resources) cannot be ruled out. Thirdly, the sample predominantly comprised tertiary hospital workers (67.3%), which may limit generalizability to primary care settings where medical-preventive integration is primarily targeted. Fourthly, monthly income was treated as a continuous variable based on category midpoint values, which may have introduced limited measurement imprecision. Fifthly, the depression screener was restricted to a 7-day recall period, capturing acute psychological distress rather than chronic longitudinal trajectories. To advance this field, future research should incorporate wider temporal assessment windows, clinical interviews, or dynamic tracking to map the long-term stability of depressive symptomology. Future longitudinal analyses from the ongoing cohort will help confirm these observed associations.

## 5. Conclusions

Our findings highlight a complex, dimension-specific association between KAP regarding medical-preventive integration and depressive symptoms among healthcare workers. The results of this study indicate that work stress is strongly associated with both behavioral engagement and depressive symptoms, suggesting that increased professional involvement may compromise mental well-being partly through elevated stress. Furthermore, monthly income plays a critical moderating role in this relationship. Compared to their lower-income counterparts, healthcare workers with higher socioeconomic resources were better able to leverage knowledge and positive attitudes into psychological benefits, while also showing a stronger positive association between behavioral engagement and depressive symptoms. Given these findings, multi-level interventions must be tailored directly to this empirical pattern. While organizational support should sustain the protective benefits of knowledge and attitude, administrative policies must targetedly mitigate the strain of practical reform engagement. When workload, role expectations, and organizational support are inadequately managed, intensive behavioral involvement inevitably escalates occupational stress and depressive symptomology, necessitating structurally optimized workload allocation and clear role boundaries to safeguard healthcare workers’ well-being.

## Figures and Tables

**Table 1:** Sample characteristics.

	*n* (Mean)	% (SD)	Range
**Knowledge**	12.32	3.60	[4, 20]
**Attitude**	14.82	3.03	[4, 20]
**Practice**	5.93	1.73	[2, 10]
**Depressive Symptoms 2024**	9.36	6.10	[0, 30]
**Work stress**	23.46	6.33	[7, 35]
**Monthly Income (thousand RMB)**	10.33	5.35	[2, 21]
**Gender**			
Female	3315	56.11	
Male	2593	43.89	
**Age**			
18~29	776	13.31	
30~39	2845	48.16	
40~49	1607	27.20	
50~59	640	10.83	
60 years old and above	40	0.68	
**Marital status**			
Married	4797	81.20	
Unmarried	1111	18.80	
**Ethnicity**			
Han Chinese	5816	98.44	
Ethnic minorities	92	1.56	
**Political status**			
Communist Party of China (CPC) member	2313	39.15	
Probationary CPC member	54	0.91	
Communist Youth League member	402	6.80	
Member of a democratic party	296	5.01	
Non-partisan individual	153	2.59	
Non-affiliated individual	2690	45.53	
**Education level**			
Secondary vocational school or below	11	0.19	
College diploma	167	2.83	
Bachelor’s degree	3263	55.23	
Master’s degree	2036	34.46	
Doctoral degree or above	431	7.30	
**Professional title**			
None	194	3.28	
Primary title	1776	30.06	
Intermediate title	2100	35.55	
Associate senior title	1220	20.65	
Senior title	618	10.46	
**Hospital level**			
Tertiary hospital	3977	67.32	
Secondary hospital	1460	24.71	
Primary hospital	471	7.97	

**Table 2:** Multiple linear regression analyses of the associations between medical–preventive integration KAP and depressive symptoms (*N* = 5908).

Variables	Model 1		Model 2		Model 3	
	B	95%CI	B	95%CI	B	95%CI
**Knowledge**	−0.074 *	(−0.134, −0.015)	0.003	(−0.001, 0.007)	−0.028	(−0.084, 0.027)
**Attitude**	−0.467 ***	(−0.531, −0.403)	−0.036 ***	(−0.041, −0.032)	−0.3	(−0.359, −0.241)
**Practice**	0.648 ***	(0.527, 0.770)	0.359 ***	(0.259, 0.492)	0.3	(0.188, 0.414)
**Work stress**	–	–	0.206 *	(0.044, 0.368)	0.198 *	(0.036, 0.360)
**Monthly Income (thousand RMB)**	–	–	–	–	−0.043	(−0.134, 0.048)
K*Income	–	–	–	–	−0.040 ***	(−0.061, −0.019)
A*Income	–	–	–	–	−0.028 ***	(−0.046, −0.012)
P*Income	–	–	–	–	0.066 **	(0.022, 0.109)
**Gender** (Ref: Female)						
Male	−0.872 ***	(−1.182, −0.562)	−0.054 ***	(−0.077, −0.030)	−0.071 ***	(−0.096, −0.045)
**Age** (Ref: 18~29)						
30~39	0.495	(−0.175, 1.165)	0.016	(−0.032, 0.064)	0.034	(−0.018, 0.087)
40~49	−0.028	(−0.822, 0.765)	−0.009	(−0.066, 0.050)	0.009	(−0.054, 0.071)
50~59	−0.635	(−1.555, 0.284)	−0.046	(−0.114, 0.022)	−0.033	(−0.108, 0.041)
60 years old and above	−3.921 ***	(−5.474, −2.368)	−0.182 *	(−0.337, −0.028)	−0.228 **	(−0.061, −0.040)
**Marital status** (Ref: Married)						
Unmarried	0.282	(−0.211, 0.773)	0.022	(−0.015, 0.059)	0.019	(−0.020, 0.059)
**Ethnicity** (Ref: Han Chinese)						
Ethnic minorities	−0.565	(−1.680, 0.550)	−0.004	(−0.097, 0.088)	−0.009	(−0.109, 0.904)
**Political status** (Ref: Communist Party of China (CPC) member)						
Probationary CPC member	−0.658	(−2.051, 0.735)	0.018	(−0.103, 0.139)	0.011	(−0.120, 0.142)
Communist Youth League member	0.039	(−0.764, 0.842)	0.056	(−0.002, 0.115)	0.031	(−0.032, 0.094)
Member of a democratic party	0.359	(−0.325, 1.042)	0.037	(−0.019, 0.092)	0.023	(−0.036, 0.084)
Non-partisan individual	0.486	(−0.451, 1.422)	0.037	(−0.037, 0.110)	0.052	(−0.028, 0.132)
Non-affiliated individual	0.406 *	(0.056, 0.754)	0.027	(−0.000, 0.053)	0.023	(−0.006, 0.052)
**Education level** (Ref: Secondary vocational school or below)						
College diploma	−1.856	(−5.701, 1.989)	−0.164	(−0.439, 0.110)	−0.166	(−0.463, 0.131)
Bachelor’s degree	−0.767	(−4.556, 3.022)	−0.119	(−0.388, 0.151)	−0.101	(−0.392, 0.190)
Master’s degree	−0.276	(−4.080, 3.525)	−0.094	(−0.365, 0.176)	−0.058	(−0.350, 0.235)
Doctoral degree or above	−0.751	(−4.582, 3.080)	−0.129	(−0.402, 0.144)	−0.099	(−0.395, 0.196)
**Professional title** (Ref: None)						
Primary title	−1.013 *	(−1.937, −0.088)	−0.038	(−0.107, 0.031)	−0.039	(−0.114, 0.037)
Intermediate title	−0.763	(−1.754, 0.227)	−0.048	(−0.122, 0.027)	−0.034	(−0.114, 0.037)
Associate senior title	−1.175 *	(−2.250, −0.101)	−0.071	(−0.152, 0.009)	−0.061	(−0.149, 0.027)
Senior title	−1.519 *	(−2.686, −0.351)	−0.086	(−0.175, 0.003)	−0.081	(−0.178, 0.015)
**Hospital level** (Ref: Tertiary hospital)						
Secondary hospital	0.719 **	(0.297, 1.140)	0.022	(−0.001, 0.054)	0.055	(0.021, 0.089)
Primary hospital	1.560 ***	(0.877, 2.243)	0.064 *	(0.015, 0.114)	0.093	(0.040, 0.146)
**Con.**	14.639 ***	(10.692, 18.587)	0.232	(−0.049, 0.513)	0.930 ***	(0.630, 1.230)

Note. Model 1 included KAP variables and covariates; Model 2 additionally adjusted for work stress; Model 3 further included income and interaction terms. Covariates were included in all models; * *p* < 0.05, ** *p* < 0.01, *** *p* < 0.001.

## Data Availability

The data presented in this study are available on request from the corresponding author. The data are not publicly available due to privacy restrictions.

## Supplementary Materials

The following supporting information can be downloaded at: https://www.mdpi.com/article/10.3390/healthcare14131832/s1, Table S1: CES-D-10 scale; Table S2: Medical-Preventive Integration Knowledge-Attitude-Practice (KAP) scale; Table S3: Work Stress Scale; Table S4: Multiple linear regression analyses.
