# Supplementary material for: Income Differences in the Association Between Medical-Preventive Integration KAP and Depressive Symptoms: A Cross-Sectional Study Among Chinese Healthcare Workers
Source: Healthcare (Basel). 2026 Jun 24;14(13):1832. doi: 10.3390/healthcare14131832 (PMC13361509; doi:10.3390/healthcare14131832)
Supplement: Supplementary file 1 [file healthcare-14-01832-s001.zip › healthcare-4298374-supplementary.pdf]

Supplementary Materials

Table S1. CES-D-10 scale

Variable	Item
1	I was bothered by little things.
2	I had trouble keeping my mind on what I was doing.
3	I felt depressed.
4	I felt that everything I did was an effort.
5	I felt hopeful about the future. (reverse)
6	I felt fearful.
7	My sleep was restless.
8	I was happy. (reverse)
9	I felt lonely.
10	I could not get going.

Table S2. Medical-Preventive Integration Knowledge-Attitude-Practice (KAP) scale

Dimension	Variable	Item
Knowledge	1	I am familiar with the concept of Medical-Preventive Integration (MPI).
	2	I understand the roles and responsibilities of hospitals under the MPI policy framework.
	3	I am aware of the specific measures implemented by my hospital in response to the MPI policy.
	4	I believe that MPI reform can help establish a people-centered, full-cycle health service system.
Attitude	1	I support the implementation of MPI reform in hospitals.
	2	I agree that hospitals should provide MPI-related training programs.
	3	I support the development of information systems to facilitate MPI reform.
	4	I agree with the mutual recognition of medical examination results across hospitals under MPI reform.
Practice	1	How frequently do you engage in MPI-related work activities in your daily practice?
	2	How frequently do you participate in MPI-related training programs?

Table S3. Work Stress Scale

Variable	Item
1	My work has a direct impact on my physical health.
2	I experience a high level of stress at work.
3	I continue to feel irritable or tense even after finishing my work.
4	My health condition would improve if my workload were reduced.
5	My work negatively affects my sleep quality.
6	I feel nervous before major surgeries or other important work-related events.
7	I find myself thinking about work even when engaged in other activities.

Table S4. Multiple linear regression analyses (N = 5908)

Variables	Model 0	
	B	95%CI
**Gender**		
(Ref: Female)		
Male	-1.1632***	(-1.480, -0.847)
**Age**		
(Ref: 18~29)		
30~39	0.409	(-0.242, 1.060)
40~49	-0.152	(-0.932, 0.628)
50~59	-1.034*	(-1.952, -0.116)
60 years old and above	-4.330***	(-6.415, -2.263)
**Marital status**		
(Ref: Married)		
Unmarried	0.251	(-0.242, 0.744)
**Ethnicity**		
(Ref: Han Chinese)		
Ethnic minorities	-0.701	(-1.945, 0.544)
**Political status**		
(Ref: Communist Party of China (CPC) member)		
Probationary CPC member	-0.708	(-2.337, 0.921)
Communist Youth League member	-0.050	(-0.832, 0.732)
Member of a democratic party	0.275	(-0.471, 1.021)
Non-partisan individual	0.581	(-0.413, 1.574)
Non-affiliated individual	0.453*	(0.092, 0.815)
**Education level**		
(Ref: Secondary vocational school or below)		
College diploma	-1.894	(-5.593, 1.804)
Bachelor's degree	-0.975	(-4.606, 2.656)
Master's degree	-0.369	(-4.012, 3.274)
Doctoral degree or above	-0.761	(-4.443, 2.920)
**Professional title**		
(Ref: None)		
Primary title	-1.110*	(-2.046, -0.175)
Intermediate title	-0.789	(-1.799, 0.221)
Associate senior title	-1.256*	(-2.348, -0.165)
Senior title	-1.694**	(-2.894, -0.494)
**Hospital level**		
(Ref: Tertiary hospital)		
Secondary hospital	0.792***	(0.371, 1.214)
Primary hospital	2.014***	(1.273, 2.758)
**Con.**	17.075***	(13.409, 20.741)

Note. Model 0 included control variable; *p < 0.05 **p < 0.01 ***p < 0.001.
